# Associations between teacher training and measures of physical literacy among Canadian 8- to 12-year-old students

**DOI:** 10.1186/s12889-018-5894-7

**Published:** 2018-10-02

**Authors:** Barbi Law, Brenda Bruner, Sara M. Scharoun Benson, Kristal Anderson, Melanie Gregg, Nathan Hall, Kirstin Lane, Dany J. MacDonald, Travis J. Saunders, Dwayne Sheehan, Michelle R. Stone, Sarah J. Woodruff, Kevin Belanger, Joel D. Barnes, Patricia E. Longmuir, Mark S. Tremblay

**Affiliations:** 1 School of Physical and Health Education, Nipissing University, 100 College Dr, North Bay, ON P1B 8L7 Canada; 2 Department of Kinesiology, University of Windsor, Windsor, ON N9B 3P4 Canada; 3 Centre for Sport and Exercise Education, Camosun College, Victoria, BC V8P 5J2 Canada; 4 Department of Kinesiology and Applied Health, University of Winnipeg, Winnipeg, MB R3B 2E9 Canada; 5 Department of Applied Human Sciences, University of Prince Edward Island, PEI, Charlottetown, C1A 4P3 Canada; 6 Faculty of Health, Community and Education, Mount Royal University, Calgary, AB T3E 6K6 Canada; 7 School of Health and Human Performance, Dalhousie University, Halifax, NS B3H 4R2 Canada; 8 Healthy Active Living and Obesity (HALO) Research Group, Children’s Hospital of Eastern Ontario Research Institute, Ottawa, ON K1H 8L1 Canada

**Keywords:** Physical literacy, Teacher, Elementary school, Physical education

## Abstract

**Background:**

Quality physical education (PE) contributes to the development of physical literacy among children, yet little is known about how teacher training relates to this development. We assessed the association between teacher training, and the likelihood that children met recommended achievement levels for components of physical literacy as defined by the Canadian Assessment of Physical Literacy (CAPL).

**Methods:**

Canadian children (*n* = 4189; M = 10.72 years, SD = 1.19) from six provinces completed the CAPL. Logistic regression was used to examine the relationship between teacher training (generalist/PE specialist), adjusting for children’s age and gender, and physical competence protocols (sit and reach, handgrip, plank, Progressive Aerobic Cardiovascular Endurance Run [PACER], body mass index [BMI], waist circumference, Canadian Agility and Movement Skill Assessment [CAMSA]), the four CAPL domain scores, and the total CAPL score.

**Results:**

Teacher training, in addition to children’s age and gender, explained only a very small proportion of variance in each model (all R^2^ < 0.03). Children taught by a generalist were less likely to reach recommended levels of motivation and confidence (OR = 0.83, 95% CI, 0.72–0.95) or CAMSA scores (OR = 0.77, 95% CI, 0.67–0.90), even when accounting for a significant increase in CAMSA score with age (OR = 1.18, 95% CI, 1.12–1.26). All other associations between measures of components of physical literacy and teacher training were not significant.

**Conclusions:**

While teacher training is hypothesized to contribute to the development of physical literacy among elementary school students, the observed effects in this study were either small or null. Children taught by PE specialists were more likely than those taught by generalists to demonstrate recommended levels of motivation and confidence, and to have better movement skills, which are hypothesized to be critical prerequisites for the development of a healthy lifestyle. Further research with more robust designs is merited to understand the impact of teachers’ training on the various components of physical literacy development.

## Background

Approximately 67% of Canadian children and youth do not obtain the recommended levels of daily physical activity (PA) [1]. Despite the numerous health-related benefits [2] and the positive impact of PA on academic achievement and cognitive outcomes [3], “physical education continues to be treated as expendable” [4] and as a marginalized subject [5] within the education system. For example, in 2016 the provincial government of Ontario, Canada dedicated $60 million to provide three math specialists in every elementary school and mandated 60 min of math per day [6]. In contrast, more than half of elementary schools in Ontario report having no physical education (PE) teacher; for those that do, it is unclear how “PE teacher” is defined (e.g., specialization, workload) [7]. This disparity in government investment came despite the fact that the proportion (33%) of children and youth achieving the guidelines for PA [1] is much lower than the proportion of elementary students meeting or exceeding the expectations for math (50% grades 1–3; 63% grades 4–6) [8]. Compare those numbers with the proportion of children who meet the expectations for reading (72% grades 1–3; 81% grades 4–6) and writing (74% grades 1–3; 80% grades 4–6) [8]. In addition, the government-mandated amount of activity per day for children in elementary school is 20 min of moderate to vigorous PA (MVPA) during instructional time, as part of Ontario’s daily PA (DPA) policy [9]; however, this requirement may or may not be included in the health and PE curriculum instruction. This highlights a notable inequality, such that schools are often mandated to provide protected time for a small number of subject areas (e.g., 20% total instructional time for math), whereas there is little or no protected time for other subject areas (e.g., 7% total instructional time for DPA). Taken together, current policies may inform perceptions that these are not valuable areas for student achievement and well-being.

One often-cited area of concern is the qualifications of teachers delivering PE, particularly at the elementary school level [10–12]. A PE specialist has been defined as a teacher who has majored or minored in PE in addition to completing a Bachelor of Education (BEd) degree, or who has received specialized and intense PE training during pre-service education [11], whereas a teacher with no specific PE training (apart from general PE courses required for all students in some BEd programs) is often considered a generalist. However, additional criteria may be applied (e.g., a certain number of years of teaching experience in PE) to meet provincial or school board designations of ‘specialist.’ The Canadian Fitness and Lifestyle Research Institute’s (CFLRI) 2015 examination of PA in elementary, middle, and secondary schools found that overall, 62% of schools in Canada have a policy to hire teachers with qualifications to teach PE and promote PA; however, of those schools, only 42% fully implemented the policy [13]. While specific data by grade levels were not provided, schools that included middle grades (i.e., grades 7–8) and those that consisted primarily of secondary grades (i.e., grades 9–12) were more likely to have a policy for hiring teachers with physical and health education qualifications [13]. This suggests that elementary schools are over-represented among Canadian schools that do not have a policy to hire teachers with PE-specific qualifications.

Advocates for the increased use of PE specialists, specifically at the elementary school level, argue that teachers with specialized training in this area will deliver higher-quality PE programming. Some international research supports this contention; for example, PE specialists offered more tasks and practice trials during PE [14] and included more PA time within PE lessons [15] than did non-specialist teachers. At present, there appears to be limited research that objectively explores this proposition within the Canadian context, with no clear consensus in terms of what aspects of PE are enhanced by specialists. For example, Faulkner et al. [16] found no difference between specialists and generalists in Ontario when it came to the number or length of PE classes per week or the amount of MVPA provided in PE. However, Mandigo et al.’s [11] study of teachers in Alberta revealed that PE specialists teaching grades 4 to 6 devoted more time to PE in their timetables than did generalists. Further, specialists reported significantly higher levels of knowledge, confidence, and enjoyment in teaching PE compared to generalists [11]. Overall, research exploring the effect of PE teacher qualifications on PE delivery and student outcomes in the Canadian context has been mainly self-reported in nature. Therefore, there is a need to examine teacher training in the context of objective student outcomes, such as measures of physical literacy.

In Canada, physical literacy is defined as “the motivation, confidence, physical competence, knowledge and understanding to value and take responsibility for engagement in physical activities for life” [17]. While it is acknowledged that physical literacy is the foundation for developing the skills, knowledge, and attitudes necessary for long-term sport and PA participation [18], the influence of exposure to teachers with PE training on children’s development of physical literacy is unknown. Although school-based PE is not the only way to develop and enhance physical literacy, schools reach a heterogeneous population and have the potential to have a significant impact on all children, particularly those who are not afforded the opportunity to develop their physical literacy through extracurricular PAs such as organized sport, or who lack support from their families or communities for PA engagement.

Therefore, the objective of this study was to assess the association between teacher training in PE (i.e., PE specialist vs. generalist) and measures of components of children’s physical literacy as assessed by the Canadian Assessment of Physical Literacy (CAPL). Specifically, we were interested in whether teacher training was associated with children’s likelihood of reaching recommended levels for overall physical literacy, as well as its components, based on the achievement categories outlined within CAPL [19].

## Methods

### Study design & sample recruitment

Between 2014 and 2017, Canadian children aged 8–12.9 years were invited to participate in the Royal Bank of Canada (RBC)–Learn to Play CAPL study. This cross-sectional, national surveillance study assessed the physical literacy levels of 10,034 children across 11 Canadian cities: Victoria, British Columbia; Calgary, Alberta; Lethbridge, Alberta; Winnipeg, Manitoba; Windsor, Ontario; North Bay, Ontario; Ottawa, Ontario; Trois-Rivières, Québec; Halifax, Nova Scotia; Antigonish, Nova Scotia; and Charlottetown, Prince Edward Island. The Ottawa site (Children’s Hospital of Eastern Ontario Research Institute) served as the coordinating centre for the overall project.

### Participants

Our primary interest was the influence of teacher training on children’s physical literacy; therefore, only children recruited through elementary schools where teacher credentials were known are included in this sample. This analysis is based on a subset (*n* = 4189, 51% girls; M_age_ = 10.72 years, SD = 1.19) of the data from the larger RBC Learn to Play–CAPL study, and only includes data from the following sites: Victoria, British Columbia; Calgary, Alberta; Winnipeg, Manitoba; Windsor and North Bay, Ontario; Halifax, Nova Scotia; and Charlottetown, Prince Edward Island.

### Measures

Demographic information (i.e., gender, age) was self-reported by children as part of the CAPL questionnaire. Children’s physical literacy was measured using the CAPL protocol established by Longmuir et al. [19]. The CAPL assesses children’s physical literacy based on a series of assessments grouped into four domains: Physical Competence, Daily Behaviour, Motivation and Confidence, and Knowledge and Understanding. The CAPL domain of Physical Competence included assessments of strength (handgrip), muscular and cardiovascular endurance (plank and Progressive Aerobic Cardiovascular Endurance Run [PACER] 20-m shuttle run), body composition (height, weight, waist circumference), flexibility (sit and reach), and motor skill proficiency (Canadian Agility and Movement Skill Assessment [CAMSA]) [20]. The Daily Behaviour domain included assessments of self-reported PA and sedentary time (CAPL questionnaire) as well as objective PA (7-day pedometer step counts). The Motivation and Confidence domain was assessed using a self-report questionnaire with items related to benefits and barriers to PA; social comparison of PA; and adequacy in, and predilection for, PA [21]. The Knowledge and Understanding domain was assessed through a self-report questionnaire, with items developed based on provincial PE curriculum documents across Canada [22]. For example, children were asked to complete multiple-choice questions asking them to indicate the maximum amount of screen time recommended for children their age, and to select the correct definition for terms within the health curriculum (e.g., cardiorespiratory fitness, muscular strength or endurance).

The composite physical literacy score is out of 100 and reflects the sum of the four domain scores. Each domain score is the sum of the component scores of each measure within that domain. For example, the Daily Behaviour domain score is based on the sum of the pedometer steps component score, the total screen time component score, and the weekly time spent in MVPA score. The scoring system allows for missing data such that a CAPL score can still be calculated if participants do not complete one measure within a domain or are missing one whole domain score. In the case of missing data, scores from other measures within the same domain are re-weighted to calculate the domain total. Within the current study, 26% of the participants were missing pedometer data. As a result, their Daily Behaviour scores are based solely on their responses to the self-report questions. Specific details on how missing data are dealt with, and a comprehensive explanation of the scoring, can be found in the online CAPL manual [23].

Overall CAPL scores are assigned to one of four categories for interpretation: beginning (children have not yet achieved a level of physical literacy comparable to most peers); progressing (improved physical literacy score comparable to most peers but not yet at the recommended level); achieving (obtained a score believed to be reflective of sufficient physical literacy); and excelling (demonstrated a high level of physical literacy). The same categories are used for each of the CAPL domains (Daily Behaviour, Physical Competence, Knowledge and Understanding, Motivation and Confidence) and for individual physical competence measures (body mass index [BMI] z score, waist circumference, handgrip strength, PACER, sit and reach, plank, CAMSA) [20, 21].

Generalized additive models for location, scale, and shape (GAMLSS) [24] were used to determine the CAPL scoring standards for each of the CAPL measures (beginning: < 17 centile; progressing: 17–65 centiles; achieving: > 65–85 centiles; excelling: > 85 centiles), taking into consideration any existing evidence-informed benchmarks for specific assessments [25]. Specific scoring criteria for each of these measures can be found in the CAPL manual [23].

To explore the relationships between teacher training and children’s physical literacy, the lead researchers at each site determined the teacher qualifications for the child’s current PE teacher. For sites within Ontario, this information was retrieved from publicly available directories that provide teacher qualifications (i.e., Ontario College of Teachers registry). In provinces where such a directory was not available, this information was provided voluntarily by either the divisional PE supervisor at the school board (in Manitoba) or by the individual teachers (in British Columbia, Alberta, Nova Scotia, and Prince Edward Island). Since definitions for PE ‘specialist’ may vary by region, school board, or school, the following criteria were used for the current study: a PE-trained teacher was any teacher with an undergraduate degree in PE, human kinetics, or kinesiology, or who had PE listed as a basic qualification (i.e., obtained during a teacher training program) and/or PE specialist as an additional qualification (i.e., obtained after graduating from a teacher training program); these represent teachers who have advanced PE training beyond the minimum requirement in a BEd program. In contrast, a generalist teacher was defined as any teacher who did not have any additional PE training based on post-secondary education, basic teaching qualification, or additional qualifications.

### Procedures

Data collection occurred between May 2014 and February 2017, and followed the RBC Learn to Play–CAPL study protocol [19]. Research staff from the coordinating centre trained personnel from all sites during a two-day workshop in Ottawa, Ontario. After training was complete, the coordinating centre obtained initial approval from the Children’s Hospital of Eastern Ontario Research Institute Research Ethics Board, and each site subsequently received approval from their corresponding institution and local school board(s). School boards sent study information to schools; interested principals or teachers contacted the researchers directly. All children invited to participate in the RBC Learn to Play–CAPL study had written consent from their parent or guardian, and provided their verbal assent before data collection began. During assessment sessions, children were assigned a non-identifying participant number. Children were asked to complete all assessments, but could opt out of any assessments they did not want to complete. Following completion of the CAPL assessments, children were thanked for their participation. Parents/guardians were provided with an individualized report on their child’s results. Schools and/or school boards were provided with reports on their children’s aggregate results.

### Data treatment

Raw participant data for each of the CAPL measures were entered into the CAPL online database, which was managed by the coordinating centre. Upon completion of each site’s data collection efforts, sites were required to manually re-enter 5% of their data for data quality assurance. A summary report was generated by each site and sent to the coordinating centre outlining the number of errors observed, and all errors were corrected. The overall data entry error rate for the RBC Learn to Play–CAPL study was 0.005%.

To address the main study aims, a series of logistic regression analyses were conducted to explore whether teacher training (accounting for children’s gender and age) was associated with children’s categorization for overall physical literacy, results for individual domain scores, or individual measures within the Physical Competence domain. Interpretation categories were combined such that children were grouped based on whether they met (i.e., achieving or excelling) or did not meet (i.e., beginning or progressing) recommended levels of physical literacy. Analyses were conducted using R 3.4.1 (R Foundation for Statistical Computing, Vienna, Austria) [26] and IBM SPSS Statistics Version 25 (IBM, Armonk, NY) [27] with a statistical significance level set at *p* < 0.05. The proportion of variance explained by each model (R^2^) is also reported to provide context for the potential practical significance, or lack thereof, of the observed effects [28, 29].

## Results

### Descriptives

Overall, 66% of the sample was taught by a teacher who had advanced PE training (*n* = 2761), with the remaining 34% (*n* = 1428) taught by generalist teachers. The proportion of PE-trained teachers to generalists varied considerably across data collection sites (Table 1).

**Table 1:** Percentage of sample from each data collection site taught by a physical education specialist

Site	Percentage of PE specialists
Calgary, Alberta	82
Charlottetown, Prince Edward Island	95
Halifax, Nova Scotia	99
North Bay, Ontario	48
Windsor, Ontario	15
Winnipeg, Manitoba	100
Victoria, British Columbia	0

Note: Percentages are not meant to be representative of population level data but reflect the teacher characteristics within specific schools included in this sample

*PE* physical education

Based on children’s scores on individual measures within the CAPL, they were categorized as beginning, progressing, achieving, or excelling within each component [23]. Table 2 shows the means for all dependent variables of interest for the total sample, as well as the two teacher categories.

**Table 2:** Means (standard deviations) for RBC Learn to Play–CAPL scores

Variable	Total (n = 4189)	Specialists (*n* = 2761)	Generalists (*n* = 1428)
Overall CAPL score (score/100)	62 (12)	63 (12)	61 (12)
Daily Behaviour (score/32)	18 (7)	18 (7)	18 (8)
Physical Competence (score/32)	20 (4)	20 (4)	19 (4)
BMI z score	0.60 (1.3)	0.57 (1.3)	0.66 (1.2)
Waist circumference (cm)	68 (10.6)	69 (10.5)	67 (10. 8)
Handgrip (kg)	34 (9.8)	35 (9.5)	32 (10.0)
Sit and reach (cm)	28 (8.4)	28 (8.7)	28 (7.7)
Plank (sec)	62 (43.7)	62 (43.1)	63 (44.8)
PACER (laps)	23 (14.6)	23 (14.1)	23 (15.4)
CAMSA(score/28)	21 (3.8)	21 (3.7)	20 (3.9)
Motivation and Confidence (score/18)	12 (2)	13 (2)	12 (2)
Knowledge and Understanding (score/18)	12 (2)	12 (2)	12 (2)

*BMI* body mass index, *CAMSA* Canadian Agility and Movement Skill Assessment, *CAPL* Canadian Assessment of Physical Literacy, *PACER* Progressive Aerobic Cardiovascular Endurance Run, *RBC Learn to Play–CAPL* Royal Bank of Canada Learn to Play – Canadian Assessment of Physical Literacy

Use of GAMLSS models [24] for establishing cut-offs for each of the physical literacy measures based on pre-determined percentiles resulted in the majority of children not meeting the recommended levels for physical literacy, with 65% of children generally being classified into the beginning or progressing categories for overall CAPL score, the domain scores, and the individual physical competence measures. The exception to this was the body composition measures, with 62% of children and 59% of children in the achieving or excelling categories for BMI z-score and waist circumference, respectively. Table 3 shows the categorization of children into the four interpretation levels for the total sample, as well as the proportion in each category according to teacher training.

**Table 3:** Classification of children into CAPL categories for the total sample (n = 4189) and proportionally by teacher training level

Variable	Beginning			Progressing			Achieving			Excelling		
	Total %	Spec. %	Gen. %	Total %	Spec. %	Gen. %	Total %	Spec. %	Gen. %	Total %	Spec. %	Gen. %
Overall CAPL score	16.4	15.6	17.8	50.0	51.4	51.8	18.4	18.5	18.1	13.7	14.4	12.3
Daily Behaviour	16.0	15.4	17.2	59.1	59.4	58.5	14.0	14.1	13.9	10.8	11.1	10.4
Physical Competence	15.1	14.6	15.9	51.0	50.7	51.4	17.7	18.0	17.2	16.2	16.6	15.5
BMI z score	14.3	14.4	16.9	1.1	1.3	0.6	21.6	22.2	20.4	62.1	62.2	62.0
Waist circumference	19.2	18.7	20.3	19.2	19.5	18.7	52.0	51.2	53.5	9.6	10.7	7.5
Handgrip	14.0	14.0	14.2	41.5	41.1	42.3	21.7	22.1	20.8	22.8	22.8	22.7
Sit and reach	17.7	18.2	16.6	46.8	45.4	49.4	20.5	20.3	20.9	15.1	16.1	13.0
Plank	12.3	12.0	13.0	57.1	58.1	55.1	16.5	16.8	16.0	14.1	13.2	16.0
PACER	14.4	12.3	18.6	51.7	54.2	47.0	17.5	17.9	16.7	16.2	15.5	17.6
CAMSA	14.0	12.2	17.3	54.1	53.1	56.0	16.8	17.2	15.8	15.2	17.5	10.9
Motivation and Confidence	16.1	15.9	16.3	49.3	48.2	51.5	18.8	18.9	18.6	15.8	16.9	13.7
Knowledge and Understanding	15.8	15.7	16.0	47.4	47.4	47.3	19.0	19.6	17.8	17.8	17.3	18.8

Notes: *Spec*. specialist (n = 2761); *Gen*. generalist (n = 1428)

Children meet recommended levels for a measure if they meet the criteria for either “Achieving” or “Excelling” interpretation categories of the CAPL. Within each category, the values for specialist and generalist reflect the proportion of children in that category taught by teachers with each level of training

*BMI* body mass index, *CAMSA* Canadian Agility and Movement Skill Assessment, *CAPL* Canadian Assessment of Physical Literacy, *PACER* Progressive Aerobic Cardiovascular Endurance Run

### CAPL domains

Logistic regression analyses showed that Motivation and Confidence was the only CAPL domain where teacher training was significantly associated with being in the achieving/excelling category, (χ^2^(1) = 6.80, *SE* = 0.07, *p* < 0.05), with students taught by a generalist being less likely to be classified as achieving/excelling (OR = 0.83, 95% CI, 0.72–0.95). Model details can be found in Table 4 for the overall CAPL score as well as the other CAPL domains (i.e., Daily Behaviour, Physical Competence, Knowledge and Understanding).

**Table 4:** Logistic regressions examining teacher training and children’s gender and age influences on whether children meet or do not meet recommended physical literacy levels for CAPL domain scores

	Classification accuracy	R^2^	B	SE	Z ratio	*p*	OR (95% CI)
*CAPL total score model*							
Intercept	67.9%	0.001	−0.66	0.32	4.11	0.042	0.518
Teacher			−0.12	0.07	2.90	0.089	0.88(0.76–1.02)
Child gender			0.06	0.07	0.76	0.384	1.06(0.93–1.20)
Child age			−0.01	0.03	0.06	0.805	0.99(0.94–1.05)
*Daily Behaviour model*							
Intercept	75.1%	0.005	0.02	0.35	0.00	0.949	1.02
Teacher			−0.11	0.08	2.06	0.151	0.89(0.76–1.04)
Child gender			**−0.15**	**0.07**	**4.49**	**0.034**	**0.86(0.75–0.99)**
Child age			−0.09	0.03	9.18	0.00	0.91
*Physical Competence model*							
Intercept	66%	0.002	−0.95	0.32	8.75	0.003	0.39
Teacher			−0.06	0.07	0.66	0.415	0.94(0.82–1.09)
Child gender			−0.11	0.06	3.04	0.081	0.89(0.78–1.01)
Child age			0.03	0.03	1.35	0.246	1.03(0.98–1.09)
*Knowledge & Understanding model*							
Intercept	63.2%	0.008	−2.06	0.32	41.72	0.00	0.13
Teacher			0.09	0.07	1.49	0.221	1.09(0.95–1.25)
Child gender			0.03	0.06	0.29	0.589	1.03(0.91–1.17)
Child age			**0.14**	**0.03**	**22.99**	**0.00**	**1.15(1.08–1.21)**
*Motivation & Confidence model*							
Intercept	65.4%	0.003	−0.25	0.32	0.60	0.438	0.78
Teacher			**−0.19**	**0.07**	**6.80**	**0.009**	**0.83(0.71–0.95)**
Child gender			0.07	0.06	1.06	0.304	1.07(0.94–1.21)
Child age			−0.03	0.32	0.60	0.438	0.78(0.91–1.02)

Note. For all models, the reference categories for teacher and gender were generalist and boys, respectively. Values for variables that are significant contributors to the model are bolded

*CAPL:* Model Χ^2^(3) = 3.67, *p* < 0.05. *Daily Behaviour:* Model Χ^2^(3) = 14.15, *p* < 0.05. *Physical Competence:* Model Χ^2^(3) = 5.92, *p* > 0.05. *Knowledge and Understanding:* Model Χ^2^(3) = 23.64, *p* < 0.05. *Motivation and Confidence:* Model Χ^2^(3) = 7.95, *p* < 0.05

*CAPL* Canadian Assessment of Physical Literacy

### Physical competence

To further explore whether teacher training influenced specific protocols within the CAPL, logistic regression models were created for each of the measures within the Physical Competence domain. CAMSA was the sole measure where teacher training (χ^2^(1) = 11.68, *SE* = 0.07, *p* < 0.05) was significantly associated with meeting recommended proficiency levels. Age (χ^2^(1) = 31.49, *SE* = 0.03, *p* < 0.05) was also a statistically significant contributor to this model. Being taught by a generalist teacher was associated with decreased odds (OR = 0.77, 95% CI, 0.67–0.90) of being classified as achieving/excelling, while older children were at greater odds (OR = 1.18, 95% CI, 1.12–1.26) of being classified as achieving/excelling. All other Physical Competence measures and teacher training associations revealed null findings; specific details for each of the models (i.e., sit and reach, PACER, handgrip, plank, BMI z score, and waist circumference) can be found in Table 5.

**Table 5:** Logistic regressions examining teacher training and children’s gender and age influences on whether children meet or do not meet recommended levels for Physical Competence measures

	Classification accuracy	R^2^	B	SE	Z ratio	*p*	OR (95% CI)
*Sit and reach model*							
Intercept	64.4%	0.003	−0.28	0.32	0.79	0.375	0.75
Teacher			−0.12	0.07	2.62	0.105	0.89 (0.77–1.03)
Child gender			**−0.17**	**0.06**	**7.03**	**0.008**	**0.84 (0.77–1.02)**
Child age			−0.02	0.03	0.39	0.530	0.98 (0.93–1.04)
*Handgrip model*							
Intercept	55.6%	0.001	−0.45	0.30	2.19	0.139	0.64
Teacher			−0.04	0.07	0.34	0.558	0.96 (0.84–1.10)
Child gender			−0.04	0.06	0.46	0.498	0.96 (0.85–1.08)
Child age			0.02	0.03	0.79	0.373	0.102 (0.97–1.08)
*PACER model*							
Intercept	66.2%	0.001	−1.12	0.32	12.16	0.000	0.33
Teacher			0.07	0.07	1.00	0.317	1.07 (0.93–1.24)
Child gender			−0.04	0.06	0.38	0.535	0.96 (0.84–1.09)
Child age			0.41	0.03	2.03	0.154	1.04 (0.98–1.10)
*Plank model*							
Intercept	69.4%	0.018	−0.81	0.33	6.01	0.014	0.44
Teacher			0.12	0.07	2.53	0.111	1.12 (0.97–1.30)
Child gender			**−0.49**	**0.06**	**52.34**	**0.000**	**0.61 (0.53–.70)**
Child age			0.02	0.03	0.32	0.574	1.02 (0.96–1.08)
*BMI z score model*							
Intercept	83.7%	0.016	2.19	0.41	28.14	0.000	8.94
Teacher			−0.14	0.09	2.41	0.121	0.87 (0.72–.104)
Child gender			**−0.51**	**0.08**	**35.76**	**0.000**	**0.60 (0.51–.71)**
Child age			−0.02	0.04	0.36	0.550	0.98 (0.91–1.05)
*Waist circumference model*							
Intercept	61.6%	0.002	1.23	0.31	15.47	0.000	3.42
Teacher			−0.09	0.07	1.50	0.220	0.92(0.80–1.05)
Child gender			−0.01	0.06	0.02	0.884	0.99(0.87–1.12)
Child age			**−0.07**	**0.03**	**5.78**	**0.016**	**0.93(0.88–0.99)**
*CAMSA model*							
Intercept	68%	0.020	−2.49	0.34	54.65	0.000	0.08
Teacher			**−0.26**	**0.07**	**11.68**	**0.001**	**0.77 (0.67–0.90)**
Child gender			−0.02	0.07	0.05	0.815	0.98 (0.86–1.12)
Child age			0.17	0.03	31.48	0.000	1.18 (1.12–1.26)

Note: For all models, the reference categories for teacher and gender were generalist and boys, respectively. Values for variables that are significant contributors to the model are bolded

*Sit and reach:* Model Χ^2^(3) = 9.91, *p* < 0.05. *Handgrip:* Model Χ^2^(3) = 2.06, *p* > 0.05. *PACER:* Model Χ^2^(3) = 2.76, *p* > 0.05. *Plank:* Model Χ^2^(3) = 54.99, *p* < 0.05. *BMI z score:* Model Χ^2^(3) = 39.21, *p* < 0.05. *Waist circumference:* Model Χ^2^(3) = 6.13, *p* > 0.05. *CAMSA:* Model Χ^2^(3) = 59.87, *p* < 0.05

*BMI* body mass index, *CAMSA* Canadian Agility and Movement Skill Assessment, *PACER* Progressive Aerobic Cardiovascular Endurance Run

## Discussion

Teacher training exhibited a statistically significant relationship with children’s CAMSA scores and Motivation and Confidence domain scores, with children taught by a specialist being more likely to meet recommended physical literacy levels than those taught by a generalist, although the effects were small. We offer some speculative thoughts on why these were the only aspects of physical literacy showing teacher effects, and why they were small. Of all the physical literacy outcomes, the CAMSA components (i.e., fundamental and complex motor skills) may be most strongly reflected in the PE learning objectives, and thus are areas that are perhaps most influenced by PE instruction. For example, interventions providing PE professional development opportunities to elementary school teachers result in enhanced PE class outcomes [15, 30, 31], such as increased time children are active in their PE classes [31, 32] and increased time spent specifically on skill drills [30]. Increased exposure time may result in greater accumulated motor skill practice among children. Perhaps the most compelling example of the effects of teacher training is the findings of the “Move It Groove It” intervention [33]. Providing generalist teachers with training on fundamental motor skills, additional resources, and support from a ‘buddy’ using a whole school approach resulted in children’s enhanced motor skill proficiency as well as an increased proportion of PE time spent in vigorous PA. However, a recent systematic review highlights that components of teacher training interventions designed to increase children’s motor skill proficiency and PA are often poorly described, and that more research is merited in this area [34].

In addition to their influence on children’s motor skill proficiency, teachers can also be important agents for fostering children’s motivation and confidence. To date, much of the research on modifying teacher behaviour to enhance students’ motivation and competency beliefs has focused on high school students; this research shows that training secondary school teachers to create environments that foster students’ sense of autonomy leads to enhanced self-determined motives and greater physical perceptions of autonomy, competence, and relatedness (i.e., psychological needs satisfaction) among their students [35]. These benefits can endure over time [36], and thus may have long-term benefits on student motivation in PE. Findings from the present work revealed that elementary school students taught by a generalist were less likely to be classified as achieving/excelling in motivation and confidence. This provides some support, albeit with small effects, for the influence of teacher training on student motivation with respect to elementary school students.

Several factors may have contributed to our finding that meeting recommended physical literacy levels (i.e., achieving or excelling) was generally not associated with teacher training. The lack of teacher effects across measures and domains, as well as the small effects on CAMSA and the Motivation and Confidence domain, may be due to several social-ecological influences, such as children’s accumulated exposure to PE-trained teachers; teachers’ professional development experiences; teachers’ years of experience; teachers’ personal PE, PA, and sport experiences; school-based policies; and available resources; as well as children’s participation in extracurricular physical activities (e.g., sport), and other family and community-based activities. Intervention research has demonstrated that while PE specialist teachers delivered higher-quality PE lessons than generalists (e.g., greater time in MVPA, fitness activities, and skill drills; teacher behaviour promoting fitness), generalist teachers who received PE training as part of an intervention also delivered higher-quality PE lessons than untrained generalists [30]. We did not assess additional teacher training and professional development, which is likely a key influence on PE delivery. Indeed, research exploring the influence of teacher training in PE suggests that many generalist teachers feel ill-prepared to effectively deliver PE [16], particularly those who have limited PA ability themselves [37].

However, it is also important to note that teacher training does not automatically imply that all teachers with training in PE deliver higher-quality PE programs than untrained generalists [38]. Teacher training programs (e.g., BEd) beyond prospective teachers’ primary undergraduate degree education, as well as teachers’ previous PE experiences, are important determinants of their confidence to deliver PE programming [37, 39]. Further, generalist teachers report that their own sport ability and personal experience with various physical activities contributes to their confidence in delivering PE [37]. While the current study used teacher qualifications as a proxy for teacher training, future in-depth research on teachers’ training from all sources (e.g., undergraduate, ongoing professional development) – as well as their personal PE, PA, sport experiences, and beliefs – is essential for understanding the complex relationship between these variables and their impact on both children’s experiences within PE settings and development of physical literacy.

Related to teacher training, school-based policies at the school, board, and/or provincial level, may also play a role in children’s PE experiences and development of physical literacy. Research supports that school culture is multi-faceted [40] and that adapting PA policies and interventions to the school context increases the implementation of PA initiatives [41]. Exploring specific school-level policies and practices was beyond the scope of this study. However, given the considerable variation in actual teacher training by data collection site within our sample (i.e., the proportion of teachers with PE training beyond what is offered as a basic requirement in the BEd program ranged from 0 to 100% by study site), we attempted to identify provincial policies on teacher qualifications. We were unable to locate provincial policies specific to PE teacher qualification requirements (aside from being a member of the College of Teachers in each province). The variation in teacher training across sites may be viewed as a limitation of our study; however, this is also consistent with existing reports that suggest most provinces do not have a policy on PE teacher training requirements [42]. The heterogeneity in teacher training across data collection sites implies that in the absence of provincial policies on PE teacher training, school boards may have developed their own formal policies and practices related to hiring ‘specialists’ for PE.

In addition, other provincial education policies may play a role in how both specialist and generalist teachers deliver PA, leading to further site variation. For example, Olstad et al. [43] reviewed DPA policies across Canada; they reported that Ontario had a moderately strong policy; Alberta and British Columbia had weak policies; and Manitoba, Nova Scotia, and Prince Edward Island did not have official DPA policies. Variations in provincial policies related to school-based PA, paired with the fact that DPA implementation [44] and PE curriculum delivery policies seem to be inconsistent across the country, suggest that school board level policies and school practices contribute to teacher practices, regardless of training.

Finally, individual factors, such as children’s participation in organized sport [45], home physical and social environment [46], play [47], and built neighbourhood environment [48, 49] can positively or negatively influence children’s development of individual physical literacy components (e.g., fitness, knowledge, daily behaviour) as well as their overall level of physical literacy. A high proportion of our sample met recommended levels for the body composition measures (i.e., 61.6% for BMI z score and 59% for waist circumference), and displayed PA levels comparable to what we would expect in a representative sample (i.e., only 24.8% met recommended daily behaviour levels). Given the large number of potential influences on children’s development of physical literacy, more research is merited to understand these complex relationships.

### Limitations and future directions

Although the current study involved a large sample size, it was cross-sectional in nature and therefore causation cannot be inferred. Further, it is unknown how much exposure students had to their current classification of teacher (e.g., taught by PE specialist for a few years or a few months). Coding of teacher training was based on the children’s *current* PE teacher, and did not consider the training of children’s previous teachers or the amount of time children had been exposed to their current teacher at the time of assessment. Therefore, we did not account for a child’s total exposure to PE-trained teachers. We recommend longitudinal surveillance of children’s physical literacy that includes their exposure to teachers who have specialized training in PE pedagogy. An in-depth examination of immediate and long-term, as well as dose-response, effects of teacher training levels on physical literacy indicators across the elementary grades (i.e., kindergarten to Grade 8) would help to identify any critical periods when children would benefit most from instruction provided by teachers with advanced PE training. For example, Canada’s Long Term Athlete Development Plan clearly identifies ages 6 to 9 years (i.e., equivalent to grades 1 to 4) as a critical period for the development of fundamental motor skills [50]. An exploration of influences on motor skill development is essential, as childhood motor skill proficiency is associated with fitness and PA behaviour in adolescence [51] as well as fitness and PA behaviour in adulthood [52]. Further, perceived competence has been identified as a mediator of the relationship between childhood motor skill proficiency and adolescent PA and fitness levels [53], and enjoyment of PE is positively associated with children’s perceived competence [54]. This highlights the importance of creating PA experiences where children can develop motor skill proficiency that also foster positive cognitions and emotions in relation to PA. Given that many young adults lack motor skill competence [55], exploration of factors that can remedy this and create positive self-perceptions is merited [53, 56].

Equally important is the need for a better understanding of school-, board-, and province-based policies around delivery of PE and other school-based PA opportunities, as well as PE professional development opportunities provided for teachers. As teachers’ confidence in delivering PE is related to their personal experiences with sport and PA as well as their prior training [37, 39], it is important to understand how these beliefs may impact their students’ own motivation and confidence in PE. Further, objective assessments are merited to identify how these factors are related to student outcomes tied to curriculum learning objectives and development of physical literacy.

## Conclusions

Overall, our data showed that teacher training had a small effect on motor skill competence and motivation and confidence, as assessed by the CAPL; all other measures and domain scores revealed null findings. The cross-sectional nature of this data, combined with a lack of research exploring the influence of teacher training on children’s physical literacy, suggests that further investigation of these relationships is warranted. Specifically, future studies should account for varying levels of teacher training in relation to PE beyond simply ‘specialists’ versus generalists and should consider the cumulative effects of children’s exposure to these teachers and its effect on physical literacy. Further study is also warranted on the role of other significant individuals (e.g., parents, coaches) on children’s development of physical literacy. Understanding the complex influences on children’s PE experiences is a first step toward ensuring all children have access to high-quality PE that facilitates their development of physical literacy.

## Abbreviations

BEd: Bachelor of Education
BMI: body mass index
CAMSA: Canadian Agility and Movement Skill Assessment
CAPL: Canadian Assessment of Physical Literacy
CFLRI: Canadian Fitness and Lifestyle Research Institute
DPA: daily physical activity
GAMLSS: generalized additive models for location, scale, and shape
MVPA: moderate to vigorous physical activity
PA: physical activity
PACER: Progressive Aerobic Cardiovascular Endurance Run
PE: physical education
RBC Learn to Play–CAPL: Royal Bank of Canada Learn to Play Canadian Assessment of Physical Literacy
